# Predictive Role of Maternal Laboratory Parameters and Inflammatory Scores in Determining Systemic Inflammatory Response Syndrome in Newborns at Birth

**DOI:** 10.3390/jpm14070672

**Published:** 2024-06-22

**Authors:** Manuela Pantea, Chaitanya Kalapala, Barkha Rani Thakur, Daniela Iacob, Claudia Ioana Borțea, Alexandra Herlo, Felicia Marc, Sonia Tanasescu, Adina Bucur

**Affiliations:** 1 Department of Neonatology, “Victor Babes” University of Medicine and Pharmacy Timisoara, 300041 Timisoara, Romania; manuela.pantea@umft.ro (M.P.); iacob.daniela@umft.ro (D.I.); bortea.ioana@umft.ro (C.I.B.); 2 Doctoral School, “Victor Babes” University of Medicine and Pharmacy Timisoara, 300041 Timisoara, Romania; 3 Katuri Medical College, Dr. YSR University of Health Sciences, Vijayawada 520008, India; kalapalachaitanya08827@gmail.com; 4 Department of Obstetrics and Gynecology, MediCiti Institute of Medical Sciences, Hyderabad 501401, India; barkharani0511@gmail.com; 5 Department of Infectious Diseases, “Victor Babes” University of Medicine and Pharmacy Timisoara, 300041 Timisoara, Romania; alexandra.mocanu@umft.ro; 6 Department of Medical Sciences, Faculty of Medicine and Pharmacy, University of Oradea, 410073 Oradea, Romania; 7 Department of Pediatrics, “Victor Babes” University of Medicine and Pharmacy Timisoara, 300041 Timisoara, Romania; tanasescu.sonia@umft.ro; 8 Department of Functional Sciences, Discipline of Public Health, Center for Translational Research and Systems Medicine, “Victor Babes” University of Medicine and Pharmacy Timisoara, 300041 Timisoara, Romania; bucur.adina@umft.ro

**Keywords:** neonatology, obstetrics, inflammation, diagnostics

## Abstract

The incidence of Neonatal Systemic Inflammatory Response Syndrome (SIRS) is a critical concern in neonatal care. This study aimed to identify maternal laboratory parameters predictive of SIRS in newborns, focusing on the establishment of diagnostic cutoffs and evaluating the predictive power of these biomarkers. This prospective cohort study was conducted from January 2023 to January 2024 across several regional hospitals specializing in neonatal care. It included 207 mother-newborn pairs, divided into groups based on the neonatal development of SIRS (66 cases) or its absence (141 controls). Key maternal parameters measured included inflammatory markers and liver enzymes, analyzed using standard biochemical methods. The study applied receiver operating characteristic (ROC) analysis to establish optimal cutoff values and conducted multivariate logistic regression to determine hazard ratios (HRs) for SIRS prediction, with adjustments for potential confounders. The study identified significant ROC/AUC values for several biomarkers. The neutrophil-to-lymphocyte ratio (NLR) demonstrated an AUC of 0.926, with a cutoff value of 3.64, achieving 81.8% sensitivity and 90.9% specificity (*p* < 0.001). The systemic immune–inflammation index (SII) showed an AUC of 0.819 and a cutoff of 769.12, with 75.8% sensitivity and 81.8% specificity (*p* < 0.001). Multivariate regression analysis highlighted that neonates with maternal SII values above this cutoff were three times more likely to develop SIRS (HR 3.09, 95% CI 2.21–4.17, *p* < 0.0001). Other notable biomarkers included dNLR and ALRI, with respective HRs of 1.88 (*p* = 0.018) and 1.75 (*p* = 0.032). These findings confirm the significant predictive value of specific maternal inflammatory markers for neonatal SIRS. These findings support the utility of these biomarkers in prenatal screening to identify neonates at increased risk of SIRS, potentially guiding preemptive clinical interventions.

## 1. Introduction

Preterm birth, defined as delivery before 37 weeks of gestation, remains a significant global health challenge [1]. It is one of the leading causes of neonatal morbidity and mortality worldwide [2]. According to the World Health Organization, approximately 15 million babies are born preterm each year, accounting for over 10% of all births globally [3]. The rates of preterm births are increasing in many countries, underscoring the need for enhanced surveillance and improved management strategies [4]. The consequences of preterm birth extend beyond the neonatal period, potentially affecting neurological development and health outcomes into adulthood [5].

Risk factors for preterm birth are multifaceted and include both maternal and fetal factors. Maternal factors such as age, history of preterm birth, multiple pregnancies, and lifestyle choices significantly contribute to the risk [6,7]. Medical conditions during pregnancy like diabetes, hypertension, and infections also play a critical role [8,9]. The complications associated with preterm births are diverse and severe, including both immediate and long-term health issues, although not associated with congenital anomalies that develop earlier in the fetal development [10,11].

Preterm labor is closely associated with systemic inflammatory processes, an association that draws significant parallels with the pathophysiological characteristics of Systemic Inflammatory Response Syndrome (SIRS) [12,13,14]. Research has increasingly indicated that the inflammatory cascades activated in SIRS may also be pivotal in the initiation and progression of preterm labor. These cascades include the release of pro-inflammatory cytokines and acute phase reactants, which can precipitate labor by altering uterine contractility and cervical integrity. In neonates, SIRS is diagnosed when at least two of the following criteria are met: core body temperature less than 36 °C or exceeding 38 °C; heart rate above 160 beats per minute for infants up to one week old, or over 150 beats per minute for those between one and four weeks old; respiratory rate above 20 breaths per minute, or the requirement for mechanical ventilation not attributable to congenital factors; and an abnormal white blood cell count, defined as below 5000 cells/mm^3^ or above 15,000 cells/mm^3^, or presenting more than 10% immature neutrophils. Understanding the interaction between preterm labor and SIRS not only illuminates the underlying mechanisms of labor but also has significant clinical implications.

Predicting which newborns are at risk of developing SIRS shortly after birth remains a challenge [15]. Traditional methods rely on clinical signs and neonatal laboratory parameters; however, these can often be non-specific and delayed. There is an emerging interest in exploring maternal laboratory parameters during pregnancy as potential early indicators of neonatal sepsis and SIRS [16,17]. Maternal biomarkers could provide insights into the in utero environment and inflammatory status that may predispose a newborn to SIRS, offering a promising avenue for early prediction and intervention.

The primary aim of our study is to evaluate the predictive role of maternal laboratory parameters in determining the risk of SIRS in newborns at birth. Specifically, we seek to identify which maternal blood markers during pregnancy correlate with increased risk of neonatal SIRS. Our objectives include determining the predictive value of these markers, establishing a potential predictive model, and assessing the feasibility of implementing such a model in clinical settings.

## 2. Materials and Methods

### 2.1. Study Design

This prospective study was conducted over a period of one year, from January 2023 to January 2024, at multiple regional hospitals with specialized neonatal care units. The primary objective was to evaluate the predictive role of maternal laboratory parameters during pregnancy in determining the risk of SIRS in newborns at birth. Participants were initially stratified into two groups based on predetermined risk levels derived from maternal laboratory parameters, including inflammatory markers. These parameters served as the exposure factor. Group 1 (SIRS) comprised pregnant women whose laboratory parameters suggested a high risk of their neonates developing SIRS, and Group 2 (No SIRS) included those with low-risk parameters. The expected effect, or primary outcome, was the incidence of SIRS in neonates, assessed at birth based on established clinical criteria.

Ethical approval for this study was granted by the Institutional Review Board of the participating hospital, ensuring adherence to the ethical standards established by the 1964 Helsinki Declaration and its later amendments, which pertain to human research ethics. The study’s protocol was thoroughly reviewed and approved, with each approval documented under specific approval numbers provided by the respective IRBs. Informed consent was obtained from all pregnant mothers before their inclusion in the study, guaranteeing their understanding and voluntary participation in the research.

### 2.2. Inclusion and Exclusion Criteria

The inclusion criteria for participating in this study were as follows: (1) pregnant patients with births complicated with neonatal SIRS; (2) mothers from whom maternal laboratory parameters during pregnancy were available, including inflammatory markers; and (3) consent for data retrieval and publication. Exclusion criteria were as follows: (1) neonates with severe congenital anomalies, particularly those affecting major organ systems such as the cardiac, pulmonary, or central nervous systems, due to their significant independent impact on morbidity and mortality; (2) neonates diagnosed with genetic syndromes that could skew the inflammatory responses typical in SIRS; (3) mothers with incomplete investigations and data necessary for this study; (4) mothers with chronic disease, active infections, GBS-positive cultures, and pregnancy complications such as gestational diabetes and gestational hypertension that could have influenced the laboratory values necessary in the current research.

For the diagnosis of SIRS within the parameters of this study, we adhered to established medical guidelines [18] that specify the necessity for at least two of the following criteria to be met: a core body temperature less than 36 °C (indicating hypothermia) or exceeding 38.5 °C (indicative of fever); a heart rate of more than 180 beats per minute in newborns up to one week old, or over 160 beats per minute in those aged one to four weeks; a respiratory rate exceeding 20 breaths per minute, or the requirement for mechanical ventilation not due to congenital reasons; and an abnormal white blood cell count, defined as below 5000 cells/mm^3^ or above 15,000 cells/mm^3^, or presenting with more than 10% immature neutrophils (band forms).

### 2.3. Biochemical Analysis

In this study, maternal blood samples were collected during routine prenatal visits and at the time of delivery to analyze various biochemical and inflammatory markers. A complete blood count (CBC), including white blood cell (WBC) count, was performed using a Sysmex XN-550 automated hematology analyzer from Sysmex Corporation, Kobe, Japan. Each sample required 1 mL of maternal venous blood collected in a tube containing ethylenediaminetetraacetic acid (EDTA) as an anticoagulant to prevent clotting and preserve sample integrity.

C-reactive protein (CRP) levels were measured using a Cobas Integra 400 Plus or Cobas e411 analyzer from Roche Diagnostics GmbH, Mannheim, Germany. These assays required 2 mL of blood collected in tubes with a separator gel to facilitate serum separation. Additional biochemical markers indicative of cellular damage and stress, such as lactate dehydrogenase (LDH) and liver enzymes aspartate aminotransferase (AST) and alanine aminotransferase (ALT), were analyzed using spectrophotometry-based biochemical analyzers.

The collection of maternal blood samples was scheduled at two critical time points, during the third trimester and immediately at delivery, in order to capture the dynamic changes in the maternal biochemical environment that could influence neonatal outcomes, particularly the development of SIRS in newborns.

### 2.4. Statistical Analysis

Data management and statistical analysis were conducted using SPSS version 26.0 (SPSS Inc., Chicago, IL, USA). Continuous variables were represented as means ± standard deviation (SD), while categorical variables were expressed as frequencies and percentages. Comparative analysis between groups (mothers of newborns with SIRS vs. without SIRS) involved Student’s *t*-test for continuous data and the Chi-square test for categorical variables. The patients were matched by gestational age.

To assess the predictive capability of maternal laboratory parameters for neonatal SIRS, receiver operating characteristic (ROC) curves were generated. The area under the curve (AUC), along with sensitivity and specificity, was calculated to determine the best cutoff values of the parameters. Multivariate logistic regression, adjusted for confounders like gestational age and maternal health conditions, was used to identify significant predictors of SIRS and calculate odds ratios. A *p*-value of less than 0.05 was considered statistically significant.

## 3. Results

The final analysis included 207 mother–newborn pairs, with 66 neonates diagnosed with SIRS and 141 without SIRS. Maternal age showed a mean of 29.42 years in the SIRS group and 29.67 years in the non-SIRS group, indicating no significant difference (*p*-value = 0.672). Similarly, the number of gestations and pregnancies reported by the mothers did not significantly differ between the two groups, with mean gestations of 2.36 for the SIRS group and 2.14 for the non-SIRS group (*p*-value = 0.153).

Regarding neonatal features, the mean gestational age at birth was slightly lower in the SIRS group at 33.14 weeks (SD ± 3.26) compared to 34.21 weeks (SD ± 4.11) in the non-SIRS group, approaching statistical significance (*p*-value = 0.064). Birth weight was also lower in the SIRS group, with a mean weight of 2436.52 g (SD ± 423.11) compared to 2507.89 g (SD ± 397.14) in the non-SIRS group; however, this difference was not statistically significant (*p*-value = 0.239). A significant finding was observed in the APGAR scores at birth. The mean APGAR score was lower in the SIRS group at 7.89 (SD ± 0.92) compared to 8.11 (SD ± 0.87) in the non-SIRS group, with this difference being statistically significant (*p*-value = 0.016), as described in Table 1.

In terms of laboratory markers, significant differences were found across multiple parameters. White blood cell (WBC) counts were higher in the SIRS group, averaging 10.27 × 10^9^/L, compared to 8.91 × 10^9^/L in the non-SIRS group, with a statistically significant difference (*p*-value = 0.007). Platelet counts were lower in the SIRS group, with an average of 248.63 × 10^9^/L versus 273.42 × 10^9^/L in the non-SIRS group, and this difference was also significant (*p*-value = 0.001). A pronounced disparity was observed in the percentages of neutrophils and lymphocytes. The SIRS group had a mean neutrophil percentage of 74.12%, significantly higher than the 59.47% observed in the non-SIRS group (*p*-value < 0.001). Conversely, lymphocyte percentages were significantly lower in the SIRS group at 19.24%, compared to 31.35% in the non-SIRS group (*p*-value < 0.001).

Further analysis revealed that lactate dehydrogenase (LDH) and C-reactive protein (CRP) levels were significantly elevated in the SIRS group, with mean values of 311.53 U/L and 12.49 mg/L, respectively, compared to 229.78 U/L and 3.24 mg/L in the non-SIRS group (both *p*-values < 0.001). Interleukin-6 (IL-6) levels were also higher in the SIRS group, with a mean of 7.34 pg/L compared to 4.98 pg/L in the non-SIRS group, denoting a significant increase (*p*-value < 0.001).

In the domain of inflammatory scores, all measures were significantly higher in the SIRS group. Notable were the neutrophil-to-lymphocyte ratio (NLR), derived neutrophil-to-lymphocyte ratio (dNLR), platelet-to-lymphocyte ratio (PLR), neutrophil-to-lymphocyte-to-platelet ratio (NLPR), AST-to-platelet ratio index (APRI), ALT-to-lymphocyte ratio index (ALRI), and the systemic immune–inflammation index (SII), all of which were significantly increased (all *p*-values < 0.001), as seen in Table 2.

The neutrophil-to-lymphocyte ratio (NLR) was significantly higher in the SIRS group, with a mean value of 3.82 compared to 2.07 in the non-SIRS group (*p*-value < 0.001). Similarly, the derived neutrophil-to-lymphocyte ratio (dNLR) was also higher in the SIRS group, recording a mean of 2.89 against 1.56 in the non-SIRS group, which was statistically significant (*p*-value < 0.001). The platelet-to-lymphocyte ratio (PLR) showed a considerable increase in the SIRS group with a mean value of 194.37, compared to 131.47 in the non-SIRS group, reflecting a significant disparity (*p*-value < 0.001). The neutrophil-to-lymphocyte-to-platelet ratio (NLPR) further underscored this trend, with the SIRS group exhibiting a higher mean of 60.29 versus 38.74 in the non-SIRS group, indicating a robust inflammatory response (*p*-value < 0.001).

Additionally, the AST-to-platelet ratio index (APRI) and the ALT-to-lymphocyte ratio index (ALRI) were significantly elevated. The APRI was notably higher in the SIRS group with a mean of 1.04 compared to 0.61 in the non-SIRS group (*p*-value < 0.001). The ALRI showed a similar pattern, with a mean of 1.19 in the SIRS group versus 0.73 in the non-SIRS group, underscoring the heightened inflammatory state (*p*-value < 0.001). The systemic immune–inflammation index (SII) was significantly higher in the SIRS group, with a mean value of 840.27 compared to 452.48 in the non-SIRS group (*p*-value < 0.001), as described in Table 3.

NLR was determined to have a cutoff value of 3.64, which exhibited high sensitivity and specificity at 81.8% and 90.9%, respectively. The AUC for NLR was remarkably high at 0.926, indicating excellent predictive capability (*p*-value < 0.001); however, this high AUC value was determined for very high scores. dNLR had a cutoff value of 3.07, showing slightly higher sensitivity at 89.4% but lower specificity at 80.3% compared to NLR. The AUC for dNLR was 0.918, also suggesting high diagnostic accuracy (*p*-value < 0.001). The PLR had a higher cutoff value of 203.06, with a sensitivity of 74.2% and specificity of 78.8%. The AUC for PLR was 0.816, indicating good but slightly lower predictive power than NLR and dNLR (*p*-value < 0.001).

**Figure 1:**
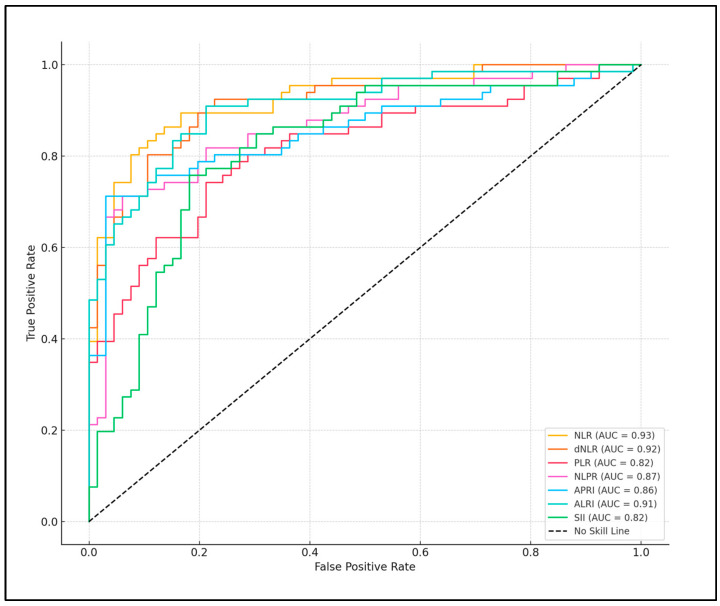
ROC curve analysis for SIRS predictors based on maternal inflammatory scores.

NLPR showed a cutoff value of 66.21, with a sensitivity of 71.2% and a high specificity of 93.9%. The AUC was 0.871, reflecting strong predictive validity (*p*-value < 0.001). APRI had a cutoff of 1.5, demonstrating a sensitivity of 71.2% and the highest specificity among the indexes at 97.0%. The AUC was 0.856 (*p*-value < 0.001). ALRI presented a cutoff value of 1.06 with the highest sensitivity at 90.9% and a specificity of 78.8%. The AUC for ALRI was 0.907, indicating high predictive efficacy (*p*-value < 0.001). Finally, the SII showed a cutoff of 769.12, with sensitivity and specificity values of 75.8% and 81.8%, respectively. The AUC was 0.819, revealing good diagnostic accuracy (*p*-value < 0.001), as seen in Table 4 and Figure 1.

NLR exhibited a significant association with SIRS development, where neonates with NLR values above the established cutoff had a hazard ratio (HR) of 2.54. This indicates that these neonates were over two and a half times more likely to develop SIRS compared to those below the cutoff, with a 95% confidence interval (CI) of 1.82 to 3.45 and a *p*-value of less than 0.0001, indicating strong statistical significance. dNLR also showed a positive association with the risk of SIRS, with a HR of 1.88, suggesting that neonates with elevated dNLR were nearly twice as likely to develop SIRS (95% CI: 1.19–2.90, *p*-value = 0.018).

PLR was found to have a HR of 1.95, indicating a near doubling of the risk of developing SIRS for values above the cutoff (95% CI: 1.26–3.12, *p*-value = 0.006). NLPR demonstrated a particularly strong association, with an HR of 2.32. Neonates with elevated NLPR values were more than twice as likely to develop SIRS (95% CI: 1.65–3.32, *p*-value < 0.0001). APRI had a HR of 2.10, again indicating a significant predictive role in the development of SIRS (95% CI: 1.58–2.95, *p*-value < 0.0001). ALRI, with an HR of 1.75, suggests that elevated ALRI values also significantly increase the likelihood of SIRS development (95% CI: 1.05–2.92, *p*-value = 0.032). Finally, the SII showed the highest HR of 3.09, indicating that neonates with SII values above the cutoff were more than three times as likely to develop SIRS (95% CI: 2.21–4.17, *p*-value < 0.0001), as presented in Table 5.

## 4. Discussion

### 4.1. Literature Findings

The study’s findings suggest a robust correlation between specific maternal blood markers during pregnancy and the risk of SIRS in newborns, highlighting the clinical potential of these biomarkers as predictive tools. Notably, the statistically significant differences in WBC counts, platelet counts, and percentages of neutrophils and lymphocytes between the SIRS and non-SIRS groups establish a clear linkage between elevated inflammatory markers and increased SIRS risk.

Further reinforcing the predictive capacity of these markers, the elevated levels of LDH, CRP, and IL-6 in the SIRS group compared to the non-SIRS group align with known pathways of systemic inflammation and cellular stress. The significant differences in these levels, particularly the marked elevation in CRP and IL-6, which are well-established acute-phase reactants, indicate an active inflammatory response in the maternal body that could potentially affect the newborn. The findings also suggest that these markers could serve as part of a broader panel to enhance the predictiveness of existing models, leading to earlier interventions.

The derived and composite inflammatory scores, such as the NLR, PLR, and the SII, further demonstrate a significant predictive capability for neonatal SIRS. Their elevated values in the SIRS group, coupled with the strong statistical significance and high AUC values in the ROC analysis, reveal their utility in not only distinguishing between potential SIRS and non-SIRS cases but also in potentially guiding therapeutic decision-making processes.

Similarly, Sofouli et al. [19] conducted a systematic review of predictive scores used for the early diagnosis of late-onset neonatal sepsis, analyzing 16 studies that incorporated original scores, validations, and mixed approaches. Their findings indicated that while these scores assist in early diagnosis, they generally exhibit limited diagnostic accuracy, highlighting a global need for a more effective and comprehensive scoring system. On the other hand, Gude et al. [20] explored the role of biomarkers in the early diagnosis, treatment, and prognosis of neonatal sepsis, emphasizing their potential to guide antibiotic usage and course duration. This study noted the promising future of biomarkers, particularly through advances in metabolomics, which could significantly enhance the management of critically ill newborns by offering high sensitivity, specificity, and predictive values.

In another study, Sofouli et al. [21] focused on the development and validation of a sepsis prediction score (SPS) based on clinical and laboratory parameters to predict septicemia in neonates in NICUs. On the day the blood culture was obtained, in the retrospective cohort, an SPS ≥ 3 predicted sepsis with 82.54% sensitivity and 85.96% specificity, and in the prospective cohort, the score predicted sepsis with 76.60% sensitivity and 72.55% specificity, demonstrating a decline in performance from the retrospective to the prospective setting. Li et al. [22] in their study, assessed the Prognostic Nutritional Index’s (PNI) effectiveness in predicting the presence and severity of neonatal sepsis. They highlighted the PNI’s clinical value, which correlated strongly with sepsis severity in neonates, suggesting its potential as a reliable predictive tool, where the ROC curve of the PNI was 0.64 (CI: 0.61–0.67) for severe sepsis and 0.69 (CI: 0.60–0.78) for septic shock. In contrast, our study focused on predefined inflammatory scores that were previously validated, obtaining higher prediction capabilities based on the AUC values that range between 0.82 and 0.91.

In their study, Wang et al. [23] analyzed 160 neonates divided into groups of mild and severe infections. Through multivariate logistic regression, they determined that significant predictors included decreased WBC, decreased platelet counts, and increased CRP levels. Their predictive models showed an area under the curve of 0.881 for decreased WBC, 0.798 for decreased PLT, 0.523 for elevated CRP, and 0.914 when these indicators were combined. Additionally, their more sophisticated models, a dichotomous variable equation model and a nomogram model, demonstrated AUCs of 0.958 and 0.914, respectively, with the nomogram model achieving a consistency index of 0.908 (95% CI [0.862, 0.954]). In comparison to our study’s findings, CRP and PLT were not tested alone for their SIRS prediction capability; however, PLT was comprised in the NLPR score, which achieved an AUC of 0.871, which is significantly higher than the result reported by Wang et al. regarding the PLT prediction alone. Conversely, Kurul et al. [24] focused on a cohort of 208 preterm neonates under 32 weeks’ gestation, analyzing 480 suspected late-onset neonatal sepsis episodes. They found that IL-6 and procalcitonin were more predictive of sepsis severity and mortality compared to CRP. Specifically, IL-6 and procalcitonin were significantly associated with 7-day mortality, with adjusted hazard ratios (aHRs) of 2.28 (95% CI 1.64–3.16) and 2.91 (95% CI 1.70–5.00), respectively. In contrast, CRP did not show a significant correlation with mortality (aHR: 1.16; 95% CI 0.68–2.00).

Liyun Xu et al. [25] and Tiewei Li et al. [26] focused on evaluating C-reactive protein and related markers for diagnosing neonatal sepsis, but with differing emphases and methodologies. Liyun Xu et al. performed a meta-analysis incorporating 31 studies involving 5698 participants to assess the diagnostic value of CRP for neonatal sepsis. They reported that CRP had a sensitivity of 0.69 and specificity of 0.77, with a positive likelihood ratio of 3.83, negative likelihood ratio of 0.38, and a diagnostic odds ratio of 12.65. The area under the curve from the summary receiver operating characteristic (SROC) curve was 0.8458, indicating a high diagnostic value. On the other hand, Tiewei Li et al. studied the C-reactive protein-to-albumin ratio (CAR) in 1076 neonates, finding that CAR was an independent risk factor for neonatal sepsis, with odds ratios of 10.144 for sepsis presence and 1.876 for severe sepsis. Their ROC analysis showed that CAR had an AUC of 0.74 for predicting sepsis and 0.70 for severe sepsis, suggesting good discriminatory power.

This study introduces a novel approach to predicting neonatal SIRS by focusing on maternal biological markers rather than the traditional neonatal biomarkers. Utilizing maternal markers offers a more accessible and non-invasive method for early intervention, enhancing neonatal outcomes. Additionally, this research pioneers the use of inflammatory scores such as dNLR, NLPR, APRI, and ALRI in this context, providing new tools for assessing maternal inflammation and its potential impact on neonatal health. These innovations streamline clinical protocols, emphasizing the utility of maternal markers as early predictors of SIRS in newborns.

The use of multiple hospitals and the stringent inclusion and exclusion criteria bolster the generalizability of the results, ensuring a diverse population sample and minimizing confounders related to neonatal and maternal health conditions that could skew the findings. However, the application of these findings in clinical practice would require the careful consideration of the logistical and the ethical implications of implementing such predictive measures, particularly in terms of the feasibility of routine maternal blood testing and the interventions that might follow elevated marker levels. These findings provide a compelling case for the incorporation of systemic inflammation markers in prenatal care protocols to better anticipate and manage neonatal SIRS, potentially improving neonatal outcomes through targeted early interventions.

### 4.2. Limitations

One of the primary limitations of this study is its reliance on a specific cohort of preterm neonates, which may limit the applicability of the findings to the general newborn population. The exclusion of neonates with severe congenital anomalies or genetic syndromes might also skew the data towards less complex cases, potentially overlooking how these variables impact the predictive validity of the biomarkers. Furthermore, the study’s observational design can introduce confounding factors that are not fully controllable, and the regional nature of the study might not capture demographic variations that could influence maternal and neonatal inflammatory responses.

## 5. Conclusions

The study conclusively demonstrates that specific composite inflammatory scores, particularly the NLR, SII, and dNLR, are highly effective predictors of SIRS in newborns. These indices, with their substantial statistical significance and high predictive values reflected in the AUC from ROC analysis, highlight their potential as crucial elements in prenatal screening protocols. The findings strongly suggest that incorporating these inflammatory scores into routine maternal health assessments could enable early identification of newborns at increased risk for SIRS, facilitating timely and targeted interventions to improve neonatal outcomes.

## Figures and Tables

**Table 1:** Maternal background characteristics and demographics.

Variables	SIRS (n = 66)	No SIRS (n = 141)	*p*-Value
Maternal features			
Age (mean ± SD)	29.42 ± 4.13	29.67 ± 3.88	0.672
Gestations (mean ± SD)	2.36 ± 0.79	2.14 ± 0.95	0.153
Pregnancies (mean ± SD)	2.97 ± 1.07	2.84 ± 1.12	0.170
C-section n (%)	41 (62.12%)	89 (63.12%)	0.889
Natural birth n (%)	22 (33.33%)	56 (39.72%)	0.377
PROM n (%)	18 (27.27%)	34 (24.11%)	0.625
Neonatal features			
Gestational age (mean ± SD)	33.14 ± 3.26	34.21 ± 4.11	0.064
Gestational weight (mean ± SD)	2436.52 ± 423.11	2507.89 ± 397.14	0.239
APGAR (mean ± SD)	7.89 ± 0.92	8.11 ± 0.87	0.016

SD—standard deviation; PROM—premature rupture of membranes; APGAR—appearance pulse grimace activity and respiration; SIRS—systemic inflammatory response syndrome.

**Table 2:** Maternal data measured at the end of third trimester.

Variables	Normal Range	SIRS (n = 66)	No SIRS (n = 141)	*p*-Value
Laboratory Markers				
WBC (×10^9^/L)	4.0–10.0	10.27 ± 2.34	8.91 ± 3.78	0.007
Platelets (×10^9^/L)	150–400	248.63 ± 55.41	273.42 ± 48.92	0.001
Neutrophils (%)	40–70	74.12 ± 8.37	59.47 ± 7.91	<0.001
Lymphocytes (%)	20–40	19.24 ± 4.89	31.35 ± 5.12	<0.001
LDH (U/L)	120–250	311.53 ± 53.21	229.78 ± 49.23	<0.001
CRP (mg/L)	<5	12.49 ± 5.72	3.24 ± 1.47	<0.001
IL-6 (pg/L)	<7	7.34 ± 3.56	4.98 ± 2.41	<0.001
AST (U/L)	10–35	32.89 ± 9.23	29.42 ± 8.77	0.009
ALT (U/L)	7–56	51.58 ± 11.45	49.72 ± 10.28	0.243
Inflammatory Scores				
NLR	-	3.47 ± 1.89	2.14 ± 1.68	<0.001
dNLR	-	2.56 ± 1.74	1.65 ± 1.59	<0.001
PLR	-	186.39 ± 42.78	129.54 ± 38.21	<0.001
NLPR	-	57.24 ± 14.82	34.68 ± 12.67	<0.001
APRI	-	0.98 ± 0.27	0.62 ± 0.21	<0.001
ALRI	-	1.13 ± 0.58	0.75 ± 0.44	<0.001
SII	-	713.32 ± 210.47	570.19 ± 195.63	<0.001

NLR—neutrophil-to-lymphocyte ratio; dNLR—derived neutrophil-to-lymphocyte ratio; PLR—platelet-to-lymphocyte ratio; NLPR—neutrophil-to-lymphocyte-to-platelet ratio; APRI—AST-to-platelet ratio index; ALRI—ALT-to-lymphocyte ratio index; SII—systemic immune–inflammation index; LDH—lactate dehydrogenase; IL—interleukin; AST—aspartate aminotransferase; ALT—alanine aminotransferase; CRP—C-reactive protein; WBC—white blood cells.

**Table 3:** Maternal data measured within 24 h from birth.

Variables	Normal Range	SIRS (n = 66)	No SIRS (n = 141)	*p*-Value
Laboratory Markers				
WBC (×10^9^/L)	4.0–10.0	9.82 ± 3.14	8.23 ± 2.07	<0.001
Platelets (×10^9^/L)	150–400	230.14 ± 60.26	276.58 ± 50.34	<0.001
Neutrophils (%)	40–70	78.37 ± 9.28	60.82 ± 8.03	<0.001
Lymphocytes (%)	20–40	17.49 ± 5.03	30.19 ± 6.17	<0.001
LDH (U/L)	120–250	325.78 ± 56.42	240.68 ± 50.78	<0.001
CRP (mg/L)	<5	13.02 ± 6.75	3.98 ± 1.52	<0.001
IL-6 (pg/L)	<7	8.38 ± 4.62	3.86 ± 2.48	<0.001
AST (U/L)	10–35	41.53 ± 10.29	30.16 ± 9.14	<0.001
ALT (U/L)	7–56	48.21 ± 12.73	31.34 ± 11.59	<0.001
Inflammatory Scores				
NLR	-	3.82 ± 1.02	2.07 ± 0.75	<0.001
dNLR	-	2.89 ± 0.81	1.56 ± 0.62	<0.001
PLR	-	194.37 ± 45.89	131.47 ± 40.35	<0.001
NLPR	-	60.29 ± 15.96	38.74 ± 13.42	<0.001
APRI	-	1.04 ± 0.31	0.61 ± 0.22	<0.001
ALRI	-	1.19 ± 0.36	0.73 ± 0.28	<0.001
SII	-	840.27 ± 220.54	452.48 ± 200.67	<0.001

NLR—neutrophil-to-lymphocyte ratio; dNLR—derived neutrophil-to-lymphocyte ratio; PLR—platelet-to-lymphocyte ratio; NLPR—neutrophil-to-lymphocyte-to-platelet ratio; APRI—AST-to-platelet ratio index; ALRI—ALT-to-lymphocyte ratio index; SII—systemic immune–inflammation index; LDH—lactate dehydrogenase; IL—interleukin; AST—aspartate aminotransferase; ALT—alanine aminotransferase; CRP—C-reactive Protein; WBC—white blood cells.

**Table 4:** Best cutoff values for SIRS prediction.

Laboratory Parameter	Best Cutoff Value	Sensitivity	Specificity	AUC	*p*-Value
NLR	3.64	81.8%	90.9%	0.926	<0.001
dNLR	3.07	89.4%	80.3%	0.918	<0.001
PLR	203.06	74.2%	78.8%	0.816	<0.001
NLPR	66.21	71.2%	93.9%	0.871	<0.001
APRI	1.5	71.2%	97.0%	0.856	<0.001
ALRI	1.06	90.9%	78.8%	0.907	<0.001
SII	769.12	75.8%	81.8%	0.819	<0.001

NLR—neutrophil-to-lymphocyte ratio; dNLR—derived neutrophil-to-lymphocyte ratio; PLR— platelet-to-lymphocyte ratio; NLPR—neutrophil-to-lymphocyte-to-platelet ratio; APRI—AST-to-platelet ratio index; ALRI—ALT-to-lymphocyte ratio index; SII—systemic immune–inflammation Index; AUC—area under curve.

**Table 5:** Regression analysis for SIRS development during the neonatal period neonates born at term.

Factors above the Best Cutoff	Hazard Ratio	95% CI	*p*-Value
NLR	2.54	1.82–3.45	<0.0001
dNLR	1.88	1.19–2.90	0.018
PLR	1.95	1.26–3.12	0.006
NLPR	2.32	1.65–3.32	<0.0001
APRI	2.10	1.58–2.95	<0.0001
ALRI	1.75	1.05–2.92	0.032
SII	3.09	2.21–4.17	<0.0001

SIRS—systemic inflammatory response syndrome; NLR—neutrophil-to-lymphocyte ratio; dNLR—derived neutrophil-to-lymphocyte ratio; PLR—platelet-to-lymphocyte ratio; NLPR—neutrophil, lymphocyte, and platelet ratio; APRI—AST-to-platelet ratio index; SII—systemic inflammation index; CI—confidence interval.

## Data Availability

Data available on request from the authors.
